# An Efficient Framework Model for Optimizing Routing Performance in VANETs

**DOI:** 10.3390/s18020597

**Published:** 2018-02-15

**Authors:** Nori M. Al-Kharasani, Zuriati Ahmad Zulkarnain, Shamala Subramaniam, Zurina Mohd Hanapi

**Affiliations:** Department of Wireless and Communication Technology, Faculty of Computer Science and Information Technolog, University Putra Malaysia, Serdang 43400, Malaysia; shamala_ks@upm.edu.my (S.S.); zurinamh@upm.edu.my (Z.M.H.)

**Keywords:** VANET Networks, stable routing, Swarm optimization, Quality of Service, network performance

## Abstract

Routing in Vehicular Ad hoc Networks (VANET) is a bit complicated because of the nature of the high dynamic mobility. The efficiency of routing protocol is influenced by a number of factors such as network density, bandwidth constraints, traffic load, and mobility patterns resulting in frequency changes in network topology. Therefore, Quality of Service (QoS) is strongly needed to enhance the capability of the routing protocol and improve the overall network performance. In this paper, we introduce a statistical framework model to address the problem of optimizing routing configuration parameters in Vehicle-to-Vehicle (V2V) communication. Our framework solution is based on the utilization of the network resources to further reflect the current state of the network and to balance the trade-off between frequent changes in network topology and the QoS requirements. It consists of three stages: simulation network stage used to execute different urban scenarios, the function stage used as a competitive approach to aggregate the weighted cost of the factors in a single value, and optimization stage used to evaluate the communication cost and to obtain the optimal configuration based on the competitive cost. The simulation results show significant performance improvement in terms of the Packet Delivery Ratio (PDR), Normalized Routing Load (NRL), Packet loss (PL), and End-to-End Delay (E2ED).

## 1. Introduction

Vehicular Ad hoc Networks (VANETs) [1] are a collection of distributed mobile nodes that are self-configured, in which each node can be a router for multi-hop relay nodes. VANETs are designed to share most features of the Mobile Ad hoc networks (MANETs), which are applied based on IEEE 802.11p technology developed to support Intelligent Transportation Systems (ITS) applications [1,2,3]. These applications are based on a context of a fully connected traffic system enabled by VANETs, where vehicles have the possibility to communicate with each other over three types; Vehicle-to-Infrastructure (V2I), Vehicle-to-Vehicle (V2V), Device-to-Device (D2D) communications, and hybrid combination of V2V and V2I [4,5]. ITS applications require a precise representation ability of routing protocols to meet the high demands of VANETs as well as QoS requirements. High movement makes the topology of Vehicular Ad-hoc Networks really short in live communication link. Further, the movement can also deteriorate routing protocols in terms of signal and message loss. These movements can negatively impact the routine of applications that have Quality of Service in terms of high responsibility, and low latency.

Adjusting routing protocols for V2V communication network in order to satisfy various QoS requirements in applications was studied in [6,7,8]. However, the natural characteristics of the vehicular network are constrained by several factors such as limited link capacity, bandwidth, and transmission range coverage. These restrictions lead to a reduced routing efficiency and an increased routing overhead [1]. The objectives of most of the current routing protocols focus on either tuning the routing parameters or adapting the efficiency of route selection to reduce network overhead. Several routing protocols were presented to utilize network resources and enhance the routing efficiency in VANETs [9]. However, most of these protocols still have shortcomings when meeting the QoS requirements and in guaranteeing the stability of network topology during the routing process. Adopting suitable routing parameters or routing functionality to enforce different QoS achieving a high level of reliability is dependent mainly on network characteristics and their constraints. A high level of communication reliability is needed to enhance traffic prioritization and optimize network resources.

Due to the high-level perspective, MANET is not suitable in VANETs, it needs to be developed to meet the specific characteristics of VANETs. Different methods to tune the routing parameters had previously been introduced to ensure a high level of performance in the dynamic network [10], yes most of them prove inadequate. Most of the them considered QoS as a major requirement to enhance the routing efficiency [11], overcomes a number of problems such as unpredictable node density [12] and the limitation of coverage [13,14]. Moreover, to address characteristics of a highly dynamic scenarios, this study can be extended by several research areas such optimization-based multiple objective which enhance delivery via D2D Communications in term of energy efficiency [5], improving the transmission capacity in dense network [15], and in a heterogeneous mobility network where the delay or disruption network tolerance presented in [16]. Most of the recent studies considered QoS to optimize the routing efficiency [17] by introducing the optimization concept [18] and Particle optimization (PSO) technique [19] in order to reduce the impact of roads constraints on routing performance [20].

Tuning routing configuration parameters is one of the most common strategies used to keep track of neighboring node status [21]. A number of studies in the literature adjusted routing configurations making it suitable and easily applied into different kinds of network [22,23]. In addition, studies tried to improve routing efficiency mainly in terms of network connectivity or available bandwidth by using multi-objective optimization strategy [22,24]. However, most of recent studies in literature succeeded somewhat in enhancing the routing performance for one or two features, but it also loses other features or part of its routing efficiency. This is because of the limitation capacity of MANETs routing protocols. Thus, the relationship should be detected between the routing performance and the influential factors of V2V system to correctly develop MANET protocols for VANET. Addressing the trade-off and balancing problem in VANET is critically required, it helps comprehending the trade-off status between QoS requirements and the routing efficiency.

In this paper, we present a framework model designed specifically to tune the routing configuration parameters in high dynamic mobility environment. This model exploits the network resources to improve the stability and reliability of routing packets between vehicles, which it index the QoS metrics as a function to balances the QoS requirements and the nature of the dynamic mobility impact. This balance reduces the trade-off between the communication cost and time needed to deliver packets successfully. In addition, we present The routing protocol for vehicular networks in urban environment called Balanced Optimized Link State Routing-Particle swarm optimization (BOLSR-PSO). This protocol is the end result obtained when the framework tune Optimized Link State Routing-Particle swarm optimization (OLSR-PSO), which improved its efficiency using the network resources by maximizing PDR, Throughput, and minimizing both NRL and E2ED.

The remainder of this paper is organized as follows; Section 2 provides an overview of the work aimed at optimizing the routing performance in dynamic topology changes. The proposed protocol and framework components are described together with the problem statement in Section 3. Section 4 introduces the optimization-based multiple objective. Section 5 presents the experiment component, analyses, and compares the results of the proposed protocol. Finally, Section 6 concludes the paper.

## 2. Related Work

The ordinary OLSR RFC-3626 [25] routing protocol was introduced to select a routing-based link state. However, this protocol has a number of weaknesses in terms of complete resource utilization and bandwidth manageability to formulating the shortest path based on the number of hop count. To enhance the reliability and routing efficiency, the authors in [26] studied the trade-off between the cost of routes, accuracy, and QoS metrics. They checked effectiveness of this solution based on utilizing of network resources by reducing the time interval between two successive beaconing packets. It offering more accurate neighbors information in spite of using different size of a Constant Bit Rate (CBR) to examine data flow. In [24] authors took into account tuning routing configuration parameters to allow each node to exchange recent knowledge periodically. This tuning was successful and immediately calculated the routing table but for less dynamic topology.

The QoS constraints do not consider the classical routing protocols when selects a path, authors in [27] studied in depth the effects of changing the internal network settings for the routing protocol performance, and illustrated the relationship between various values of path control messages on the increasing/decreasing routing overhead ratio. In addition, they studied the impact of increased data rate with node density on routing performance. Accordingly, the authors in [28] guarantee the QoS metrics in terms of the response time and packet loss. Furthermore, the QoS allowed optimizing links stability based on nodes mobility information.

A comprehensive study about the impact of data flows on achieved routes validity used CBR data flow between vehicles to study the impact of different mobility models on the performance of the routing protocol in Vehicular Networks [29]. For this propose, an interesting study included several obstacles and constraints; a QoS was used as a metric to optimize OLSR parameters in terms of packet delays and PDR by reducing the refresh interval time [30]. The authors studied different impacts on OLSR timer message intervals by tuning OLSR parameters under various node speeds and densities. On the other hand, authors in [31] took the QoS requirements to enhance routing protocols in Vehicular Networks, and highlighted the most specific challenges and limitations.

Tuning routing parameters is an important issue to enhance the exchanging route information between neighboring nodes correctly [23], and leads to improved route discovery decisions in high dynamic mobility environments. The reason for tuning any routing protocol configuration parameters was proposed to enhanced routing ability and functionality to cope with the target environments. Moreover, the main goal of optimization was to detect broken paths and to enhance the maintenance route procedure. In [32] authors focused on the influential issues for the robustness of routing protocols. They took into consideration the requirements of QoS such as high packet delivery ratio when translating videos streamed over User Datagram Protocol (UDP) application. The goal of this adaptation was to improved the robustness of routing protocol when network topology changes and no transmission mechanism was applied on higher layer.

The framework approach was introduced to obtain the knowledge about the current mobility as a main issue in dynamic mobility, which used the video-streamed base on UDP in order to optimize routing decisions and reduce the influence of path losses [33]. In [11], the authors used optimization techniques to tune the routing parameters in order to adjust routing functionality and find the best solution based on a fitness cost, which ensures routing packets to its final destination. The new parameters significantly improved the routing performance in terms of end-to-end delay compared to the default parameters. They use Ant Colony Optimization to select the path at crossroad intersections, which enhances the network connectivity based on the tuned routing functionality pertaining some of the QoS metrics to adopt forward packet mechanisms.

For a self-optimizing scheme to balanced routing resources in wireless sensor networks, the authors used Ant Colony Optimization to utilize and optimize network resources [34]. Most of these methods are adopted to enhance the paths with the best quality function, leading to a reduced packet loss, energy usage, and extended network lifetime. The disadvantages of these methods, however, is that the selection of the route is improved based on only two function qualities: the hop-count, and path quality. Another interesting study was proposed based on the automatic tuning method [35], which used a number of techniques to optimize the transmission time. The performance of the proposed protocol with the new configuration was compared to human configuration under different urban and highway scenarios. A heuristic method was used to tune the routing protocol parameters obtaining the configurations automatically to deal with the specific characteristic of VANETs [22]. However, this tuning only emphasized on enhancing the routing configuration parameters in terms of delay metric, only, without taking into consideration the remaining QoS metrics such as throughput.

In summary, The primary objective of this work is to define the best parameter values for the routing protocol in VANET environments. These values are obtained by sensing the best response time for any change in the topology network, especially in terms of the overhead and end-to-end delay.

## 3. Framework Components

The framework model should offer a comprehensive view of network environment as close as possible to the real world environment. This model is carried out by different stages and procedures which interact automatically to obtain efficient routing configurations. In order to obtain fully the information of vehicles network communications, each scenario is run to compute the communication cost of the whole vehicles connectivity in term of PDR, throughput, NRL, and E2ED. This is done through network simulation which link OLSR with IEEE 802.11p and VANET scenarios. Then, the connectivity cost is calculated as a cost function. This cost is used as a main factor in PSO algorithm to generate an optimized solution (Configuration of parameters).

### 3.1. OLSR Protocols and Parameter Overview

A link state routing protocol was proposed in [25] for discovering the shortest path. This protocol has an efficient mechanism that can be adapted to sensing and collecting information about its neighbors and immediately create a route based on the number of hops. Each node generates a different type of message in order to sense its neighbor nodes [24]. Basically, the nodes exchange recent knowledge periodically based on HELLO-interval and Topology Control (TC) messages in order to declare all the links. Then, each node collects links’ information of one and two-hop neighborhood in order to update their routing tables and look for the candidate nodes that have a high probability to become a MPR node.

In OLSR, the MPRs are selected using the HELLO-interval messages and are only responsible for forwarding TC messages to special nodes in the network. This feature ensures a minimum level of packet broadcasting over the whole network. Therefore, the performance of OLSR is greatly influenced by the number of HELLO-interval and TC messages and their interval time. Furthermore, increasing the rate of Hello-interval and TC messages leads to more frequent updates in the topology table thereby resulting in more routing overhead [27]. The HELLO-interval represents the time interval in seconds needed to exchanged information about neighbor nodes, where each node knows the expected time of each link. Accordingly, the REFRESH-interval minimizes the routing overhead by controlling the number of HELLO-interval messages. Thus, the adjustment of the OLSR message interval should be taken into consideration to reduce the overhead and to enhance network scalability.

This work tunes the most important configuration parameters, which have a great impact on network performance as proposed in the ordinary OLSR. Table 1 summarizes the main configuration parameters of OLSR RFC-3626. For this purpose, it is necessary to declare all the links with all neighbors at least once within the scope of the updated REFRESH-interval. Consequently, the HELLO-interval must be smaller than or equal to the REFRESH-interval. The TC-interval represents the volume of information that may be involved in the periodic HELLO-interval message. By exchanging HELLO-interval messages, each node can receive information from the broadcast of the TC-interval messages throughout the whole network. The messages contain a set of fully advertised links between nodes, which must be compiled to determine the routing table. The NEIGHB HOLD TIME parameter refers to the time validity of the information through the hello default configuration, which should be equal to 3 HELLO-interval. The TOP HOLD TIME is the parameter that indicates how much time is required as a validity period for the information in the TC interval message. The default configuration is equal to 3 TC-interval. Nodes in OLSR-RFC network determine its willingness value between 0 to 7 to act as a MPR and participate in forwarding based on their optimal mode of source destination. DUP HOLD TIME value of (30 s) was calculated according to the time that node needs to process the information about forwarded packets. Therefore, we choose this value because it is more compatible with the other BOLSR-PSO configuration parameters.

As a result, these parameters define the degree of time allocated, which can be selected from a ready-made group of eight levels for re-routing message information that is equal to 3 by default. The interesting features of OLSR [25] made it the best candidate to tune the configuration of VANETs environment. The simple operation and the MPR set concept allow this protocol to offer very competitive delays which is an important factor in VANET application.

### 3.2. PSO Heuristic Algorithm Overview

PSO is an adaptive algorithm developed by Dr. Eberhart in [36]. The main concept of PSO is to emulate the social behavior of animal societies without any leader. This approach uses a computational method to optimize a problem by using a set of particles that move in a specific area and share knowledge about position and velocity update [37]. The topology of movement allows all particles to communicate with each other with few simple formulas, it moves and defines a subset of particles to communicate and exchange information about their own best known position as well as the entire swarms’ in the search-space. The particles simultaneously achieve their best condition through communication, and the best solutions are obtained by repeatedly moving and exchanging information until the best condition is discovered.

In order to initialize the population, we need to define the group of *i*-th in particle $X_{i}$ (solutions) where *i* = i1, i2, …, in represents the number of particles and *j* = j1, j2, …, jm refers to the *j*-th dimension of the problem in the domain of the search space. These particles are distributed randomly in the search space, and the optimal solution is made depending mainly on updating the particles position with each iteration.

$X_{i}j\left(t\right)$: The location of particle *i* according to the *j*-th dimensional component.
$V_{i}j\left(t\right)$: The velocity of particle *i* according the *j*-th dimensional component.
$B_{i}j{\left(t\right)}^{local}$: The best location (local best) of particle *i* according to the *j*-th dimensional component.
$B_{i}j{\left(t\right)}^{global}$: The global best location of the swarm according to the *j*-th dimensional component.

The basic operation of PSO is more appropriate to solve dynamic mobility problem, and it is used to mange the network resources [21]. To find an optimal solution, the set of particles $X_{i}j$ should communicate and exchange available information about the network topology in order to update the local best $B_{ij}^{local}$ and global best $B_{ij}^{global}$ for each particle. Accordingly, the parentage of position change for each particle *i* is presented as $V_{i}$ = $v_{i}1$, $v_{i}2$, …, $v_{i}j$. The topology of movement allows all particles to communicate with each other according to few simple formulas, in which all particles have converged to a point in the search-space to create a sequence of solutions. In such cases, the cost function must be optimized to provide suitable solution for each particle. thus, the set of solutions are required to control the direction of movement for each particle at each iteration in the search space. Let *f* denote the fitness function needed to be optimized. Then the local best of particle *i* can update its velocity and position at iteration t as.

(1) $$X_{ij}^{new-best}=X_{ij}+V_{ij}^{new-best}$$

where $V_{ij}$ is velocity of particle *i* at *j*-th dimensional.

(2) $$V_{ij}^{new-best}=W_{i0}\;V_{ij}+Rnd_{1}\;W_{i1}\;\left(B_{ij}^{local}-X_{ij}\right)+Rnd_{2}\;W_{i2}\;\left(B_{ij}^{global}-X_{ij}\right)$$

where $W_{i0}$, $W_{i1}$, $W_{i2}$ represent the acceleration coefficients that are applied in the beginning process and denote a positive constant weight for each particle in the swarm.

$W_{i}0$ – The inertia weight conditioning term.
$W_{i}1$ – The memory weight conditioning term.
$W_{i}2$ – The cooperation weight conditioning term.

The $W_{i0}$ and $V_{ij}$ in the formula refer to the behavior of the particle movement where $W_{i}1$ represents the best position stored in memory. The $W_{i}2$ represent the communication enhancement between the verticals. More information about PSO algorithm can be found in [19,38].

## 4. Optimization-Based Multiple Objective

### 4.1. Performance Index Model

**Figure 1:**
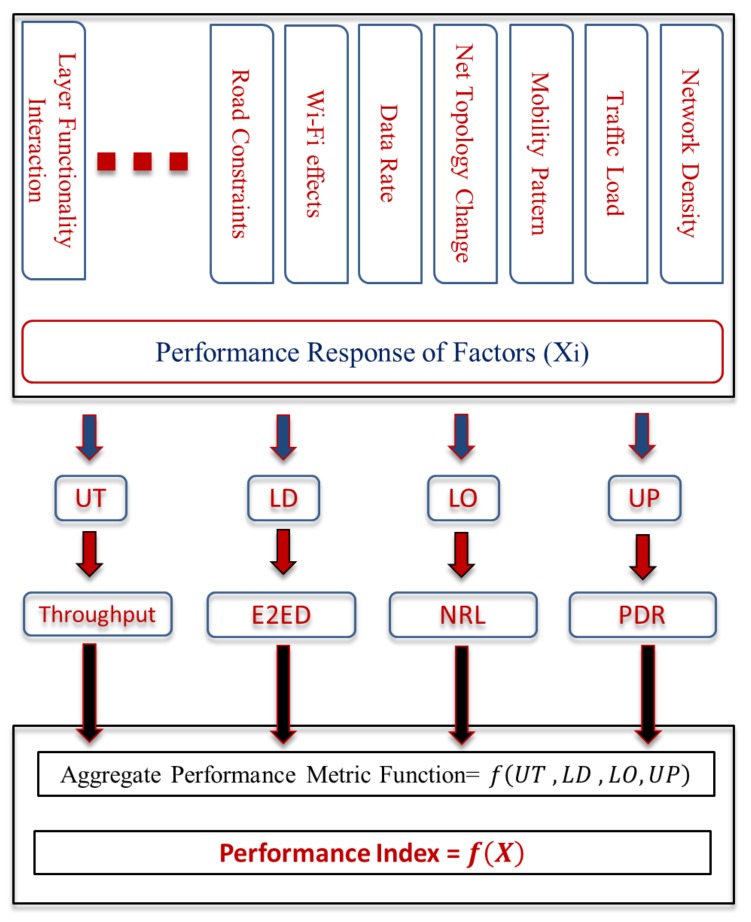
Statistical Function of Performance.

To detect a fit configuration for routing protocol, we introduce a reliable and robust communication cost function to aggregate the weighted cost of each factor in the network to a single scalar value. This value function has been defined to maximize the network connectivity during the path discovery phase and to reduce the routing overhead. Our approach can take the advantage of the communication capabilities among vehicles to provide an accurate status for different urban scenarios. The overall routing performance is influenced by numerous factors such as the degree of node mobility patterns, network density, traffic load, frequent topology changes, and wireless channel effects. Moreover, this function balances the trade-off level between the QoS requirements and the routing limitations during the route discovery mechanism, which achieves the best overall performance in respect to each performance metric. The advantage of this strategy is its ability to independently examine the different metrics for a variety of urban scenarios. In addition, the proposed model provides an efficient method for comparing two or more effective factors, which index the set of response metrics in one function, as shown in Figure 1.

The performance model represents arithmetically the weighted response of each performance metric, comparing the communication cost with the respective upper and lower bounds of each factor. In the literature, a number of studies address the routing configuration problems in the high dynamic network, however, none of these studies considered balancing routing performance according to the nature of VANET environment. This work maps factors of performance metrics to a single function based on balancing the trade-off between the QoS constraints and the degree of dynamic mobility. This model is carried out by coupling two different stages: an optimization procedure and a simulation stage. We considered Throughput, Packet Delivery Ratio (PDR), End-to-End Delay (E2ED) and Normalized routing Load (NRL) as the main QoS factors to calculate the communication cost (fitness function).

Let WT,WP,WD and WO refer to the weight vectors that index Throughput, PDR, E2ED and NRL factors, respectively. In this case, the four metrics are equally important, and the weight value assigned to each metric is 0.25. The main reason behind assigning a weight value for each metric is to give an accurate priority for each metric in order to increase its sensitivity to detect any change in network topology [39]. The quality of communication then can be defined as $U_{T},\;U_{P},\;L_{D}$, and $L_{O}$, in which $U_{T}$ represents the throughput metric and $U_{P}$ represents the PDR metric. $U_{T}$ and $U_{P}$ values must be maintained as high as possible. On the other hand, the $L_{D}$ and $L_{O}$ metrics refer to E2ED and NRL respectively, in which they must be as low as possible. When evaluating the factors’ value of $U_{T},\;U_{P},\;L_{D}$, and $L_{O}$ metrics, the effectiveness of the formula should express the distribution of these response metrics. This is to offer a high level of individual ability to each metric and their global influence on the network performance.

To define the best-fit formula that is able to reflect the correct trade-off status between the OLSR configuration and frequent change in network topology, we need to identify the appropriate QoS constraints to control the values for each metric. Let $V_{T,i};V_{P,i};V_{D,i};V_{O,i}$ be the values that seek to maximize the performance ratio of throughput and PDR, and minimize the ratio value of E2ED and NLR, respectively. These factors are used to balance the values for the trade-off status between the QoS requirements and rapid topology exchange in urban scenarios. Therefore, we construct the function of the communication cost as follows, according to the criticality of each metric:

(3) $$Communication\;cost=f(W_{T}\frac{V_{T,i}}{U_{T}},W_{P}\frac{V_{P,i}}{U_{P}},W_{D}\frac{L_{D,i}}{V_{D}},W_{O}\frac{L_{O,i}}{V_{O}})$$

where *i* refers to the interact response when there is any change in one of the varying factors – T,P,D, or *O*. Therefore, the arithmetic average of summation can be formulated as a weighted ratio of the response metrics according to the respective upper or lower bound. Where *f* represents the fitness cost of communication that quantifies overall system performance at each iteration. To define the upper or lower bounds between 0 and 1, the equation of cost function *f* can be formulated as follows:

(4) $$communication\;cost=\sqrt{\left(W_{T}\frac{V_{T,i}}{U_{T}}+W_{P}\frac{V_{P,i}}{U_{P}}+W_{D}\frac{L_{D,i}}{V_{D}}+W_{O}\frac{L_{O,i}}{V_{O}}\right)}.$$

The objective of the cost function *f* is paramount to maintain the quality of solution. The ratios of $V_{T,i}$/$U_{T}$, $V_{P,i}$/$U_{P}$, $L_{D,i}$/$V_{D}$, and $L_{O,i}$/$V_{O}$ represent the values of relevant metrics and influencing factors. For example, the ratio $V_{T,i}$/$U_{T}$ represents the measurement of how much $V_{T}$ value of response metric *i* deviates from its respective upper bound $U_{T}$. Accordingly, the $V_{P,i}$/$U_{T}$ measures the deviation value of response metric *i* from its respective upper bound $U_{P}$. Similarly, the ratios $L_{D,i}$/$V_{D}$ and $L_{O,i}$/$V_{O}$ measure the deviations of $V_{D}$ and $V_{O}$ from its lower bound $L_{D,i}$ and $L_{O,i}$, respectively. The key advantage of the performance index is to enable us to quantify the main and interactive factor values on the performance response, which characterizes the functional relationship between a set of performance metrics.

### 4.2. Configuration Optimization Architecture

The proposed framework model is comprised of three stages: The network simulation with different urban scenarios, communication cost function, and PSO optimization algorithm. These procedures generate optimal configurations of OLSR protocol for a given VANET scenario. In each urban scenario, all mobile nodes are equipped with IEEE 802.11p network interface card which give each node the ability needed to send and receive messages according to Wi-Fi constraints. This ability is conditional by several factors such as limitation of distance, bandwidth availability, link life time, directions, and the varying speed of vehicles. This is because of the routing protocol discovers the path through the vehicles based on the network topology, which build it by periodically broadcasting control messages. These messages are used to update and maintain the network topology information within specific period time. In such VANET environments, this time is not compatible with routing configuration parameter values defined in (Table 1. OLSR RFC 3626 Configurations), which lead to frequent link breakage and flooding more packets to detect the network topology, and the network topology information is often outdated. Thus, computing an optimal configuration for the parameters of this protocol is crucial before developing any routing protocol for VANET.

To build route successfully, OLSR uses eight different configuration parameters. These parameters represent the time for gathering neighbors information, processing, calculating and select routes. OLSR selects routes based on the principle of the information collection about the global state of the network which represent the critical issues in the VANETs. To ensure more reliability, and stability in the selected paths, relaying messages between nodes in the network and selecting shortest path should be smoother. For this reason, this work is seeking to tune the routing parameters of OLSR protocol efficiently. We evaluate and analyze the global simulation result of different urban scenarios by the performance index model, which provide the information of global communication. These information are used to obtain the communication cost for each performance metric.

**Figure 2:**
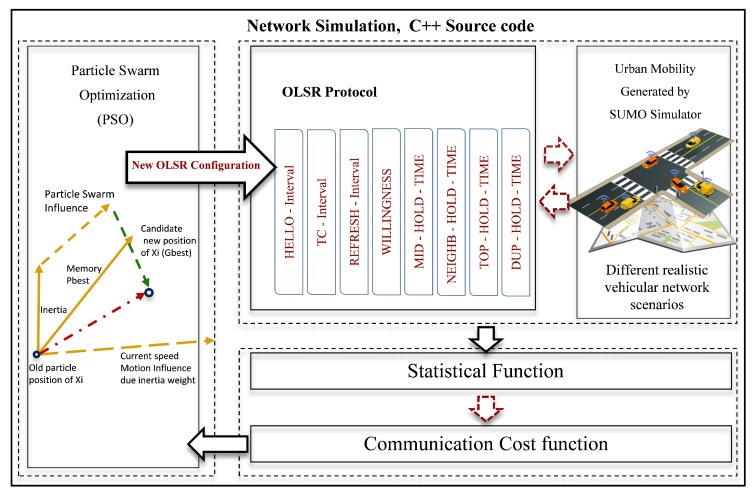
Framework Model for Optimization Routing Performance.

The proposed model index aggregates the global network information of the routing connectivity in terms of the amount of information successfully received in a time unit (Throughput), the number of packets received by destination nodes to the number of packets sent by the source node (PDR), the number of forwarded packets divided by number of data packets received (NRL), and the ratio of time difference between every packet sent and received to the total time difference over the total number of packets received (E2ED). Figure 2 describes the main components of the framework model.

In order to save network bandwidth, and improve the transmission ability of the network, the proposed equation is made to reflect correctly the deviating values of response metrics from its respective performance metric bound. The performance metrics values are placed in the equation in the form of numerator and denominator to maximize and minimize the performance metric. Additionally, the deviating values obtained from the equation reflects the balance of the trade-off between the QoS requirement and the efficiency of routing, these values plays an important role in balancing the weight of communication costs. The PSO algorithm calculates the optimal fitness value using the communication cost obtained from cost function. The communication cost is associated with each particle which affects the behavior of particles to find the global fitness value of a single parameter at a time. These values represent the new value of BOLSR-PSO configuration parameters. This process continues to happen once a new communication cost is calculated until an optimal global fitness value is obtained for each parameter. The new values rising from this process are the configuration parameters of BOLSR-PSO (Table 2).

**Figure 3:**
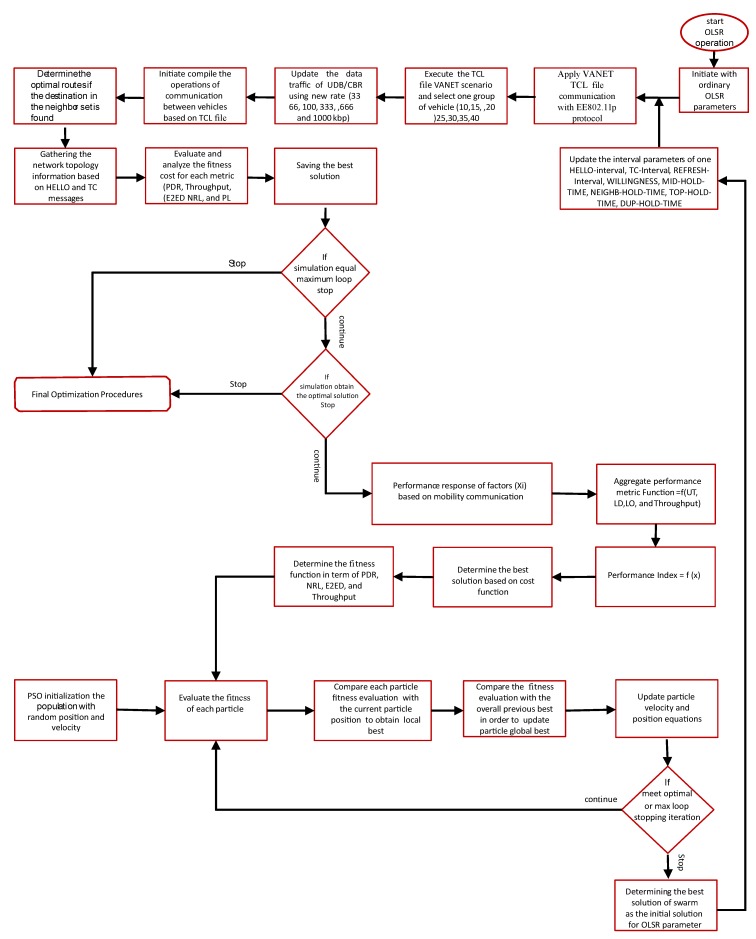
Simulation Flow Diagram of Optimization Model.

Figure 3 summarizes the model stages and presents the flow of interactions between network simulation and the other components.

## 5. Simulation and Performance Analysis

### 5.1. Define Urban Network Scenario

**Figure 4:**
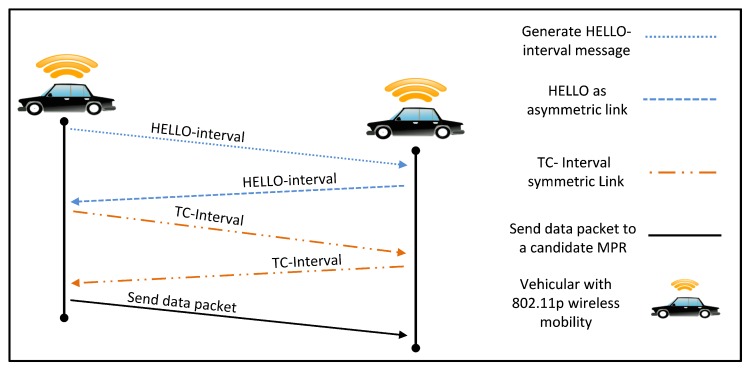
Communications Mode Between Two Pairs of Vehicles.

Nowadays, there are different approaches developed to solve the complex problem of VANET simulation. There are numbers of road traffic simulators which generate NS-2 format traces file, but the most comprehensive ones use OpenStreetMap (OSM) [39] such as Simulation of Urban Mobility SUMO [40] and VanetMobiSim simulator [41]. These combined approaches allow a direct interaction between the communication network system and the vehicular traffic dynamics to specify the different scenarios, which imports common roads information (XML code) from OpenStreetMap such as road edges, traffic lights, traffic signs, road direction, road conditions, vehicle speed on the roads, etc. The SOMU simulator generates vehicular mobility trace files that is used as input for a mobile wireless of OLSR network. In order to understand the procedures that is needed between two vehicles to create route and exchange data packet. Each vehicle broadcast HELLO-interval and TC messages to detect next hop in its communication range, a symmetric link is necessary to estimate the quality of link and then initiate transmit data packet. The procedures of communications mode between two vehicles in the VANETs uses an ad-hoc way approaches, communication range, control messages, and shortest path method, all depicted in Figure 4.

**Figure 5:**
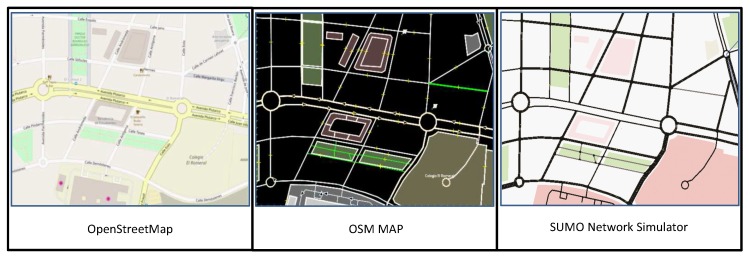
Conversion the Realistic Road Map to Traffic Mobility.

In order to obtain results close to the real world, we have defined 10 realistic vehicular mobility scenarios. This scenarios will be evaluated by an existing specific VANET simulator as suggested in [22]. The critical differences between MANETs and VANETs make most of current ad hoc protocols unsuitable to apply in high dynamic environment. The certain characteristics which make most of current ad hoc protocols unsuitable to apply in high dynamic environment. This work take into consideration the relation between the traffic in dynamic mobility and the cost communication network. Thus, we have to define 10 urban scenarios with a specific data flow in which these scenarios represent various possible connections that is presented in the network. A set of cars were defined on specific area to exchange data in an urban environment roads [22]. These vehicles moving with a fluctuating speed and directions throughout the simulation time. Figure 5 shows the conversion stages of road network that was used to evaluate the performance of the proposed method.

We take into consideration the relation between the number of network nodes and the selected area, vehicle speed movement, limit of covering range, available bandwidth, and simulation time. For example, the number of nodes should be higher in highway wireless network environments. The reason of large scale density is to reduce the likelihood of packets loss due to vehicles traveling at higher speeds than in urban areas environments. In our scenarios, there are 40 vehicles moving and exchange information data about routes in an area of 1400 m × 1200 m, and the vehicles travel on the urban scenario with varying speeds of 10–50 km/h, and the transmission power range is adjusted to 250 m. The setting of VANET scenarios are adjusted automatically by SUMO simulator.

### 5.2. Simulation Environment

In order to investigate the efficiency of our solution, we implemented and compared the OLSR-PSO [24], UM-OLSR [42], OLSR-Gomez [24], and OLSR RFC-3626 [25] protocols within the Network Simulator. For more accurate vehicular mobility patterns, this work uses the real map tool to define realistic urban environment scenarios using the freely available OpenStreetMap [39]. In particular, we use Simulation of Urban Mobility (SUMO) [40] as a tool uses to generate realistic VANETs simulations, where the nodes follow the behavior of vehicles in a road. This tool extract the most common information form the open street map such as road edges, road direction, traffic signs, road conditions, traffic lights and constraints [43]. In our simulations, we also consider a wide range of traffic densities according to the specific mobility data models proposed in [22], which performs 10 scenarios for each group of cars moving randomly in different directions through an area of 1400 m × 1200 m within three minutes as in Table 3. The movement mobility and number of cars are proportional to their corresponding selected area as suggested in [22].

The vehicles move through urban roads at varying speeds of 10–50 km/h. The transmission power range is adjusted to 250 m according to the characteristics of the network system (IEEE 802.11p) interface card [44]. The simulation is performed using an i7 processor (6 cores, 15 MB cache 3.8 GHz), 8 GB Quad channel DDR3 RAM, 2 TB hard drive, and Linux Fedora core 16. For each run, there are fixed data rates that transmit 4 packets per second. Each vehicle sends a packet in 512 bytes size. This means the vehicles exchange the data packets by the constant bit rate (CBR). Table 3 summarizes the simulation phase parameters.

In this simulation, we performed 40 independent runs of every scenario in order to ensure the accurate values of the average communication cost. This cost should be as close as possible to real world environment, where the quality of cost is evaluated using six network data loads (33, 66, 100, 333, 666, 1000 kbps) in this area. Table 4 summarizes the main parameters of the PSO algorithm that can be chosen. In order to increase the efficiency of the PSO algorithm in the search space, the particle population and the number of iterations are performed according to [19]. Furthermore, the random search algorithm (RAND) [45] is implemented to compare the efficiency of the proposed parameters. Several metrics can be used to evaluate and compare the BOLSR-PSO performance. These metrics correctly describe the average change in a response variable produced by a change in the level of a factor.

We used the most widely accepted parameters, such as average E2ED, PDR, NRL, and PL.

### 5.3. Results and Discussion

We discussed the framework proposed to augment OLSR routing protocol efficiency, which ultimately proved to balance QoS metrics. These performance metrics were studied to find the impact of different OLSR configurations on the proposed fitness function. The trade-off between accuracy of routes and cost in the proposed equation manages correctly the capacity of links between the vehicles, therefore, reducing the necessary number of packets exchanged, reducing link failure and help in avoiding congestion. Table 2 describe the configuration parameters of the proposed BOLSR-PSO with four routing protocols – OLSR RFC-3626, OLSR-PSO, OLSR-Gomez, and RAND algorithms.

In our BOLSR protocol, whenever a vehicle aggregates the neighboring vehicles information at a specific point of time, it uses HELLO-interval and TC messages to estimate the quality of communication state in the routes discovering process. It is important to note that the parameters that are used in OLSR protocol mange this process at limited time. This time must be enough to collect, process, calculate, select routes, and broadcast route information. If the time interval for each parameter was not enough to received packet during this specific period, then, the probability of link failure increases. This may lead to packet loss and increase end to end delay.

#### 5.3.1. Packet Loss

**Figure 6:**
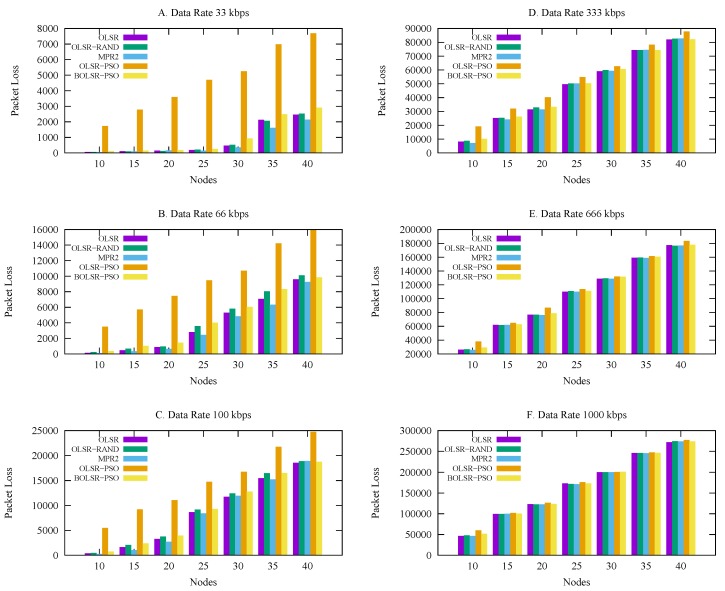
Packet Loss vs. Number of Nodes.

Figure 6 describes the impact of the proposed BOLSR-PSO configuration on the number of packets loss under different traffic loads. This result explains the advantage of the proposed strategy, which presents the relation between the size of packet loss and the increase in node density with load traffic. In low traffic loads, BOLSR-PSO shows a reasonable packet loss compared to the OLSR RFC-3626, OLSR-Gomez, and OLSR-RAND algorithms. The reason for this is that the proposed configuration is able to adjust the communication time until a link failure occurs, while the trade-off strategy works as a metric to balance the natural dynamic mobility and the efficiency of the routing functionality. The chart clearly shows that OLSR-PSO always has poorer performance than the other routing protocols under the most traffic loads, but with an increase in the traffic load and number of vehicles, it remains the same. Table 5 reflect the statistical results indicating a packet loss mean of 82,793, median 61,094, mode of 28, and the proposed algorithm achieved the best packet loss score of 28. Comparing the proposed algorithm to OLSR, BOLSR-PSO achieve a slightly better mean than OLSR. Since a higher packet loss implies a low packet delivery number, this makes the functionality of protocol generate additional packets leading to network conjunction especially in high density network.

#### 5.3.2. Packet Delivery Ratio

**Figure 7:**
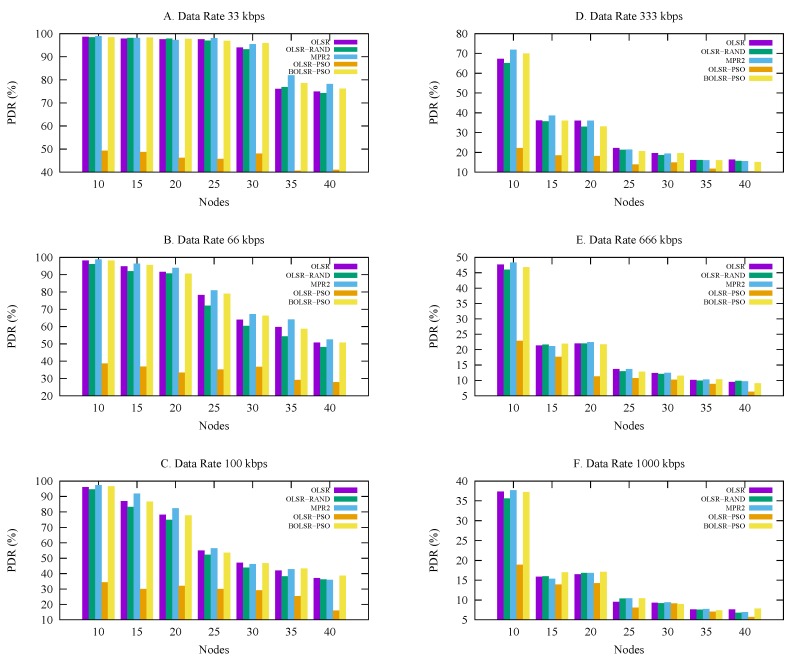
Packet Delivery Ratio vs. Number of Nodes.

Figure 7 describes the delivery ratio results using several urban scenarios for different node densities. The proposed BOLSR-PSO performed better than the OLSR RFC-3626, OLSR-PSO, OLSR Gomez and RAND algorithm. The configuration of BOLSR-PSO is suitable for coping with rapidly changing network topology. It gives the ability to deliver packet quite more than other protocols, it also reaches a good results in term of overhead and the End-to-End delay. Table 6 shows the statistical results of BOLSR-PSO and OLSR-Gomez algorithms in which they have optimal packet delivery ratio with a mean (53.3, 53.6), median (53.6, 54.1), and mode (96.9, 96.4) respectively. BOLSR-PSO have a best of 99.7 and worst of 6. Here again, most of the routing demonstrates similar results whereas the OLSR-PSO represents the worst PDR performance. However, there is a little difference between the delivery ratios achieved under different scenarios, which can be justified to the routing configuration background of each protocol.

#### 5.3.3. Normalized Routing Load

**Figure 8:**
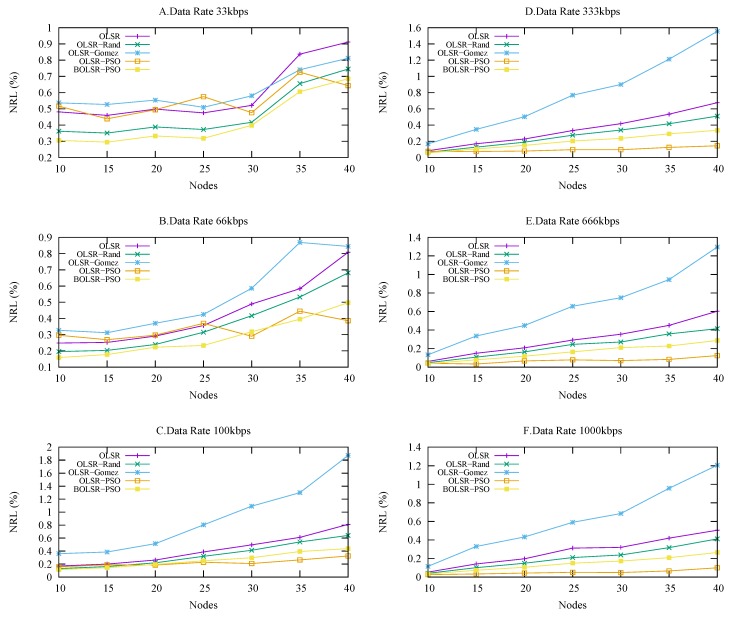
Normalized Routing Load vs. Number of Nodes.

Figure 8 shows the NRL performance against the node density with a variance in the traffic load. The proposed BOLSR-PSO and OLSR-PSO demonstrate the lowest routing load especially in high density network compared to the OLSR RFC-3626, OLSR-Gomez, and OLSR-RAND algorithms. This is because of reliable configuration parameters are obtained using PSO algorithm. Evidently, the low NRL experimented in these parameters enhanced the functionality of routing operation, so they can even reduce the possibility of network failure and avoid conjunction in high routing loads. Table 7 shows that normalized routing load results were the best compared to other algorithms excluding OLSR-PSO which was fairly bad in packet delivery ratio and the number of packet loss. Obtained mean was 220, median 189, mode 8, best 0.06 and worst 596.7. OLSR-Gomez results were fairly good in packet delivery ratio and packet loss, however, the normalized routing load results was not favorable. We noticed a slight advantage in the OLSR-PSO configuration when the vehicle density increased in high traffic load. In addition, the BOLSR-PSO respects the balancing of the trade-off model requirement, which selects a stable path according to the cost of the QoS metrics and the constraints of the network topology. Globally in routing, the lower NRL, the higher the ability to maintain routing table.

#### 5.3.4. Average End-to-End Delay

**Figure 9:**
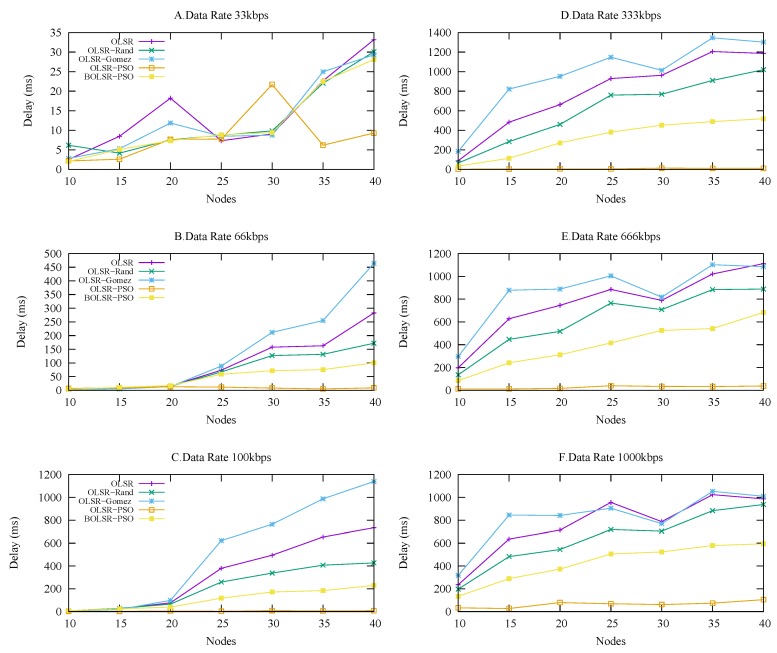
End-to-end Delay vs. Number of Nodes.

Figure 9 describes the ability of the BOLSR-PSO algorithm to deal with the frequent changes in the network topology, which performs better in dynamic mobility than the OLSR-Gomez, OLSR RFC-3626, and OLSR-RAND routing protocols. The optimal parameter values give routing functionality the time needed to send packets successfully, process, and select the path. Moreover, the optimal configuration enables the OLSR functionality to make the best use of the available bandwidth, which results in a moderate delay under different urban scenarios. Table 8 shows the statistical results of average end-to-end delay in which BLOSR-PSO was good pertaining dynamic mobility. The mean is 0.28, median 0.23, mode 0.35, best 0.009 and worst 0.95. As expected, the percentage of End-to-End delay for all the selected protocols increases with an increase in the node density. The worst results were obtained by OLSR-RFC and OLSR-Gomez protocols, which required addition time to deliver the packets. This drawback limited the routing functionality to support the most cooperative vehicular application. The reason for this problem is the lack of harmony between the time interval and the and the time required to detect the path and route packets successfully. In contrast, OLSR-PSO provides good performance due to the intelligent method that uses the delay cost function as a metric to optimize the routing operations.

#### 5.3.5. Networks with Common Characteristics

The behavior of each algorithm shows the capability of delivering, gathering, and processing route knowledge of different dynamic mobility patterns. The effectiveness of each approach is described by the functional relationship between the QoS metrics that influence the performance routing protocol and the mathematical approach. The proposed protocol help to improve the operations functionality to reduces link failures and conjunction problem by collecting accurate and up-to-date data about VANET network timely. Therefore, the proposed protocol enhances the stability and flexibility of the network by balancing between the QoS requirements and the OLSR configuration constraints. Table 9 demonstrates the summary of the main performance metrics obtained from each protocol in terms of packet loss, NRL, PDR, and E2ED. The table interprets the median results in Table 5, Table 6, Table 7 and Table 8. The median results were chosen because they are real values unlike the mean values.

## 6. Conclusions

This work introduced an ideal solution to fulfill the QoS requirements for solving optimal routing configuration problems. This solution satisfies various demands of the VANET network by adjusting the routing configuration, it also can balance different response metrics effectively which can be integrated into a single scalar value that gives a better evaluation and comparison. In an urban environment, the proposed BOLSR-PSO routing protocol is able to utilize the network resources information efficiently and provide smooth routing data packets between the vehicles. It gives better performance compared to the standard OLSR RFC-3626, OLSR-PSO, OLSR-Gomez and RAND algorithms. The simulation results showed that the proposed configuration significantly improves routing performance under different conditions of traffic density. As a future work, we plan to design scalable method that can efficiently deal with high topology change under different network condition. This work takes into account the position of relay selection nodes and Roadside Units (RSUs) to evaluating the quality of link connection.

## Figures and Tables

**Table 1:** OLSR RFC-3626 Configurations.

Parameters	RFC Standard Values	Extent of Range
HELLO-Interval	2.0 s	[1.0, ........, 30.0]
REFRESH-Interval	2.0 s	[1.0, ........, 30.0]
TC-Interval	5.0 s	[1.0, ........, 30.0]
NEIGHB HOLD TIME	3 × HELLO-Interval	[3.0, ........., 100]
TOP HOLD TIME	3 × TC-Interval	[3.0, ........., 100]
MID HOLD TIME	3 × TC-Interval	[3.0, ........., 100]
DUP HOLD TIME	30.0 s	[3.0, ........., 100]
WILLINGNESS	3	[1, 2, 3, 4, 5, 6, 7]

**Table 2:** Configuration Parameters of Each Protocol.

Parameter								
Protocol	HELLO-Int	TC-Int	REFR-Int	WILL	MID-HT	NEIG-HT	TOP-HT	DUP-HT
OLSR-RFC	2.0	5.0	2.0	3	15.0	6.0	15.0	30.0
OLSR-PSO	8.909	7.192	9.663	1	91.303	67.238	72.693	21.572
BOLSR-PSO	5.90	5.19	6.663	3	15.576	17.77	15.576	30.0
OLSR-Gomez	1.0	2.5	1.0	3	7.5	3.0	7.5	30.0
OLSR-RAND	3.730	5.188	6.188	4	34.467	5.400	40.164	31.515

**Table 3:** Simulation Parameters Settings.

Parameters	Symbol
Simulation area	1400×1200 m${}^{2}$
Number of Scenarios	10
Number of vehicles	10, 15, 20, 25, 30, 35, 40
Vehicle speed	10–50 km/h
Transmutation range	250 m
Channel type	Wireless
MAC layer type	IEEE 802.11p
Radio-propagation model	Nakagami
Routing layer	OLSR
Channel bandwidth	6 Mbps
CBR Packet Size	512 bytes
CBR Data Rate	33, 66, 100, 333, 666, 1000 kbps
CBR time	60 s
Simulation time	180 s

**Table 4:** PSO Parameters Settings.

Parameters	Value
Local Coefficient	2
Social Coefficient	2
Inertia Weight	0.50
Maximum iterations	500
Swarm size	40

**Table 5:** Statistical Result of Packet loss Performance Analysis.

	BOLSR-PSO	OLSR-RFC	OLSR-RAND	OLSR-Gomez	OLSR-PSO
Mean	82,793.8	82,879.6	84,344.4	83,036.5	86,929.6
Median	61,094.4	61,302.6	61,581.1	61,049.0	64,640.4
Mode	28.03	34.7	61,580.1	100,130.6	1704.4
Best	28.0305	34.7051	87.8117	40.2057	1704.3806
Worst	272,844.5102	270,937.9988	275,417.8391	273,434.5102	276,548.6217

**Table 6:** Statistical Result of PDR Performance Analysis.

	BOLSR-PSO	OLSR-RFC	OLSR-RAND	OLSR-Gomez	OLSR-PSO
Mean	53.3	52.8	52.6	53.6	28.2
Median	53.6	53.5	53.7	54.1	28.2
Mode	96.9	97.2	97.2	96.4	45.2
Best	99.7035	99.5027	99.6027	99.9157	51.7402
Worst	6.0021	5.7274	4.7432	4.7502	2.6205

**Table 7:** Statistical Result of NRL Performance Analysis.

	BOLSR-PSO	OLSR-RFC	OLSR-RAND	OLSR-Gomez	OLSR-PSO
Mean	220.1	388.5	325.4	416.5	36.1
Median	190.0	366.1	276.2	424.4	32.3
Mode	8.1	4.1	6.7	4.1	4.1
Best	0.06928	0.0992	1.9821	0.0972	0.0872
Worst	596.7082	1028.6217	940.3557	1058.9915	106.7772

**Table 8:** Statistical Result of Delay Performance Analysis.

	BOLSR-PSO	OLSR-RFC	OLSR-RAND	OLSR-Gomez	OLSR-PSO
Mean	0.2820	0.4380	0.3400	0.5978	0.3026
Median	0.2359	0.4022	0.3022	0.5780	0.2820
Mode	0.3522	0.3715	0.2431	1.00	0.0230
Best	0.0091702	0.0151383	0.0151383	0.0430792	0.0043541
Worst	0.9564540	0.9989901	0.9704306	1.00	0.956004

**Table 9:** Routing Performance and Experimental Output.

Protocol					
Performance Metric	BOLSR-PSO	OLSR-RFC	OLSR-RAND	OLSR-Gomez	OLSR-PSO
Packet Delivar Ratio	Good	Good	Good	Good	Not good
Packet Loss	Good	Good	Good	Good	Bad
Normalized Routing Load	Very good	Not good	Good	Bad	Very good
End-to-End Delay	Very good	Not good	Not good	Bad	Very good
